# Fluoride enigma in end stage renal disease

**DOI:** 10.6026/973206300200998

**Published:** 2024-09-30

**Authors:** Paramaturi Sharon Rose, Kurpad Nagaraj Shashidhar, Uppalamethi Munilakshmi, Muthyala Meghanath

**Affiliations:** 1 Department of Biochemistry, Sri Devaraj Urs Medical College, Kolar, Tamaka, Karnataka, India

**Keywords:** Fluoride, IL-6, diabetic nephropathy

## Abstract

Fluoride is an essential micro-mineral. Chronic exposure to fluoride affects collagen biosynthesis, insulin resistance, diabetes mellitus and kidney damage. Present prospective study, include 142 subjects categorised into group I and II. Serum samples was separated and stored appropriately. Statistical significant values were observed in urea, creatinine, calcium, magnesium, fluoride and IL6 in DN. Serum fluoride, total proteins, and calcium showed a positive correlation in group I versus group II. Fluoride is one of the key adjunct and surrogate molecule in causation of micro-vascular complications of diabetes.

## Background:

Diabetes mellitus is a metabolic disorder [1]. World Health Organization predicted prevalence of diabetes of 330 million by 2030 [2]. Diabetes results from insufficient insulin synthesis or action leading to increased glucose [3]. Uncontrolled diabetes leads to micro and macrovascular complications [3]. Diabetic nephropathy is one of the major microvascular complications and diagnosed by estimating microalbumin in urine, which is a measure of 30 to 300 mg/day [4, 5]. Fluoride is an essential micro mineral required for enzyme activities, skeletal and teeth [6]. It is the 13th most abundant element on earth and exists naturally in mono-anionic form [7]. Kolar district Karnataka, India is fluorosis endemic area with fluoride concentration beyond permissible limit of 0.4-4.6 ppm [8]. Fluorosis endemic zone is attributed to scanty rainfall and deep groundwater. Chronic exposure to fluoride from drinking water induces insulin resistance, diabetes mellitus and kidney damage [9]. World Health Organization Guideline recommends permissible fluoride in drinking water upto1 ppm [10]. Chronic exposure to fluoride affects collagen synthesis and post-translation modifications affecting the biological activity [11]. Chronic exposure to fluoride has detrimental effects on kidney such as decline in glomerular filtration rate affecting the filtration capacity of the kidney and decreased excretion of waste products [5]. Individuals with kidney diseases are at increased risk of developing soft tissue diseases, skeletal and dental fluorosis [13]. Therefore, it is of interest to find association of fluoride in non-skeletal Fluorosis.

## Materials and Methods:

## Study design:

Total number of study subjects n=142; categorised into

Group I: Individuals with Diabetic Nephropathy (n=71)

Group II: Study subjects with Diabetes (n=71)

We have taken into consideration confidence interval 95%; Margin of error 5%; and response distribution; 50%Patients visiting R. L. Jalappa hospital attached to Sri Devaraj Urs Medical College a constituent of Sri Devaraj Urs Academy of Higher Education and Research were included for the study. Study was approved by Institutional Ethics Committee vide No: SDUMC/KLR/IEC/ 557/2023-24. Blood samples were collected from the study subjects in their comfortable position. Serum was separated and stored appropriately till analysis. Estimation of urea, creatinine, albumin, total proteins, calcium, magnesium and IL-6 were analysed in Vitros 5600 and dry chemistry analyser. Serum fluoride analysis was done by ion selective electrode Thermo Fisher Scientific No A214.

## Inclusion criteria:

[1] Clinically proven type 2 Diabetes Mellitus and Diabetic Nephropathy

[2] Subjects exposed to high fluoride content

## Exclusion criteria:

[1] Gestational diabetes mellitus

[2] Individuals with Type1 Diabetes Mellitus

[3] Kidney injury secondary to poisoning, snakebite, myocardial infraction, heart failure and medications majorly affecting renal function tests.

## Variables:

Statistical skewness was analysed for the variables. Statistical analysis was done by using licensed version of SPSS 23.0 IBM

Paired student t-test and Pearson correlation was used to find the significance and p value <0.05 was consider as statistically significant.

## Results and Discussion:

Table 1, represents the comparison between Group I and Group II. We observed statistical significance and elevated levels of blood urea, creatinine, calcium, magnesium, fluoride and IL6 in DN compared to DM. Higher values are attributed to extent of renal damage which are confirmed by the values of serum urea, serum creatinine and urine microalbumin. We observed high levels of serum albumin and total proteins in DM subjects compared to DN but statistically not significant. Table 2 Depicts Pearson's correlation of fluoride with DN and DM and also independently with DN and DM. No significant correlation was observed between group I and group II. However we documented a significant positive correlation of total proteins with an r=0.32* and p=0.01 in Group I. Further with Group II there was a positive correlation of fluoride with calcium of r = 0.29* and p= 0.02. CKD is one of the major community health concerns. It is highly prevalent in developing and developed countries [14]. Around 6.3% of CKD are reported in rural parts of Karnataka, India [15]. CKD is sequelae of diabetes and hypertension. CKD may result in complications involving cardiovascular system [16]. It can be prevented in early stages with proper care against environmental factors, life style modification, avoiding smoking and alcohol consumption [17]. Fluoride being the mono-anion binds with divalent cations, post translational modification of collagen synthesis and affects the carbohydrate and protein metabolism. To what extent the epigenetics plays a critical role linking Fluoride is yet to be derived. Fluoride being the negatively charged molecule bind with Ca^+2^, Mg^2+^, K^+^ and Mn^2+^ compete at the active site of the enzymes requiring these cations resulting in altered synthesis and post translational modifications viz insulin and collagen. Calcium a divalent cation plays a critical role in lowering the blood pressure acting through angiotensin 2 receptors [18]. Impact of fluoride on calcium metabolism is the leading cause of cardiovascular events in DN. This can be attributed to hypertension either as a consequence of electrolytes imbalance or due to defective renal regulation of blood pressure [18]. Fluoride induces insulin resistance in diabetes and inhibition of enolase enzyme increase glucose levels. Increased glucose enters polyol pathway and converts into Galactitol, Sorbitol, Aldactol and other sugar Alcohols. These sugar Alcohol compounds are hygroscopic in nature and accumulates in the cytosolic compartment of the cell [9, 19]. Increased IL6 causes insulin resistance by affecting the phosphorylation of insulin receptor and insulin receptor substrate1 and induce the expression of SOCS3, a potential inhibitor of insulin signalling [20]. IL6 induced insulin resistance in adipose tissue and liver may synergize with proinflammatory cytokines to produce cell damage which is implicated in T2DM. Since T2DM is due to insulin receptor defect and insulin resistance, the role of IL6 in DM can be attributed to this [20]. Enolase inhibition by fluoride has a synergistic effect on IL6.This synergistic effect is consistent in our study IL6 values are higher in group II. IL6 signalling is also involved in the progression of DN [21]. IL6 responses are mediated via GP 130-STAT-3 dependent mechanisms which trigger the transition from innate to adaptive immune response and regulate immune cell infiltration [21]. As there is damage to glomerular basement membrane in DN; excretion of IL6 is impaired. Biological reference interval of IL6 in clinically proven healthy adults is < 5 pg/ml the higher levels observed in group I may be contributed to subclinical infection and inflammation or insulin resistance.

Despite the local population chronically exposed to high fluoride levels, the observed values of fluoride in both group I and II are within the biological reference interval. Controlled fluoride values in the blood may be contributed to present consumption of reverse osmosis water awareness programs initiated by Fluorosis Referral and Research Laboratory of our institution and Government agencies of the impact of fluoride on health. We also have observed high levels of urea and creatinine which is consistent with group I compared to group II. The albumin levels are slightly higher in group I compared to group II. However in both the groups the albumin levels are around 83% of the lower limit of biological reference interval (3.5-5 g/dL). The total proteins values are also lower in both groups which is around 55%; this reduction in albumin and total proteins may be linked to decrease synthesis or increased excretion. Calcium values are slightly higher in group I compared to the biological reference interval which may be attributed to the decrease in excretion of Ca^2+^ or Ca^2+^ binding with other negative ions or proteins. Mg^2+^ values are higher in both groups which may be linked to the kinase enzyme activity of the glycolytic pathway. We propose defluoridation of water, regular fluoride estimation of potable water and also products grown using fluoride rich water, milk from the bovine sources fed with fluoride rich water, inclusion of fluoride as a routine parameter in all prediabetic, diabetic and also at risk diabetics to ensure prevention of microvascular complications of diabetes in fluoride endemic areas. Environmentalists and Scientists working on water to concentrate on mechanisms to neutralize the filtered out water of the RO plants as it recharged back to the soil.

## Conclusion:

Fluoride is an adjunct surrogate ion for microvascular complications of diabetes mellitus.

## Figures and Tables

**Table 1:** Comparison between DN and DM

**Variables**	**Group I**	**Group II**	**p value**
	**Mean ± SD**	**Mean ± SD**	
Serum Urea (mg/dL)	126.13±60.75	24.6±13.04	0.001*
Serum Creatinine (mg/dL)	4.73±3.21	0.69±0.31	0.001*
Serum Albumin (g/dL)	2.89± 1.07	3.53±0.52	0.159
Serum Total Proteins(g/dL)	3.70±1.17	6.7±0.71	0.23
Serum Calcium (mg/dL)	13±4.06	8.87±0.64	0.001*
Serum Magnesium (mg/dL)	2.14± 0.20	1.7±0.26	0.001*
Serum Fluoride (ppm)	0.50± 0.38	0.38±0.36	0.05*
Serum IL6 (pg/ml)	6.46. ±4.57	4.1±2.4	0.03*
Group I: Diabetic Nephropathy, Group II: Diabetes, p< 0.05: Statistically significant

**Table 2:** Correlation of fluoride with biochemical variables in diabetic nephropathy versus diabetes mellitus

	**Variables**		**U**	**Cr**	**Alb**	**TP**	**Ca^2+^**	**Mg^2+^**	**IL6**	**NA**	**m Alb**	**M Alb**
Fluoride(ppm)	DN	r value	0.09	-0.06	0.06	0.32	-0.03	0	0.14	0.01	0.09	0.04
		p value	0.46	0.56	0.61	0.01*	0.84	1	0.26	0.93	0.45	0.73
	DM	r value	0.03	0.1	-0.05	-0.09	0.29	-0.01	-0.05	ND	ND	ND
		p value	0.83	0.4	0.7	0.45	0.02*	0.93	0.69	ND	ND	ND
*p < 0.05 Considered as significant. U: Urea, Cr: Creatinine, Alb: Albumin, TP: Total Proteins, Ca2+: Calcium, Mg2+:
Magnesium, IL6: Interleukin 6, NA: Normo albuminuria, m Alb: Micro albuminuria, M Alb: Macro albuminuria, ND: Not Detectable.
